# Catechins Controlled Bioavailability of Benzo[a]pyrene (B[α]P) from the Gastrointestinal Tract to the Brain towards Reducing Brain Toxicity Using the In Vitro Bio-Mimic System Coupled with Sequential Co-Cultures

**DOI:** 10.3390/molecules24112175

**Published:** 2019-06-10

**Authors:** Kang-Hyun Jeong, Hyun Jeong Lee, Tae-Sik Park, Soon-Mi Shim

**Affiliations:** 1 Department of Food Science and Biotechnology, Sejong University, 98 Gunja-dong, Gwangjin-gu, Seoul 143-747, Korea; skyou0094@naver.com (K.-H.J.); lhjung91@hanmail.net (H.J.L.); 2 Department of Life Science, Gachon University, Bokjung-dong, Sujung-gu, Sungnam-si 461-701, Gyeonggi-do, Korea; pts9918@gmail.com

**Keywords:** catechins, B[α]P, HepG2, HBMECs, blood-brain barrier, bioavailability

## Abstract

The aim of the current study was to examine the preventive effect of green tea catechins on the transport of Benzo[a]pyrene (B[α]P) into the brain using an in vitro bio-mimic system coupled with sequential co-cultures. When 72 μM of catechins was pre-treated, cellular cytotoxicity induced by IC_50_ of B[α]P in human liver hepatocellular carcinoma (HepG2) and human brain microvascular endothelial cells (HBMECs) was reduced by 27% and 26%, respectively. The cellular integrity measured in HBMECs, which was exposed to IC_50_ of B[α]P, slowly decreased. However, the pre-treatment of catechins retained cellular integrity that was 1.14 times higher than with the absence of catechins. Co-consumption of catechins reduced not only the bio-accessibility of B[α]P in digestive fluid, but it also decreased absorption of B[α]P in human intestinal epithelial cells (Caco-2) with a HepG2 co-culture system. It was found that approximately a two times lower amount of B[α]P was transported via the blood-brain barrier (BBB) compared to only the B[α]P intake. These results are taken in conjunction with each other support that catechins could be able to prevent brain toxicity induced by B[α]P in the human body by limiting the bio-availability of B[α]P.

## 1. Introduction

Polycyclic aromatic hydrocarbons (PAHs) are generally recognized as one of the environmental contaminants that significantly affect human carcinogenesis [1]. One of the PAHs, benzo[α]pyrene (B[α]P), is categorized as a Group 1 carcinogens by the International Agency for Research on Cancer [2]. Benzo[a]pyrene (B[α]P) is mainly produced from burning wood, cigarette smoking, car exhausts, and smoked or grilled food [2,3]. It is absorbed through the skin, respiration, and the intake of contaminated food [4]. It was monitored in various types of foods, such as vegetables (0.013 µg/kg), cereals (0.262 µg/kg), smoked fish (0.8 to 13.9 µg/kg), dairy products (0.078 µg/kg), and milk/milk products (0.011 µg/kg) [5]. According to a previous study, human exposure to B[α]P through food intake was about 0.125 µg per day [6]. Even though B[α]P has been identified as a carcinogenic compound even at a low exposure concentration, tolerable daily intake (TDI) has not been generally established [7].

After being absorbed in the body, B[α]P binds with the aryl hydrocarbon receptor (AHR) and is activated by diol-epoxide metabolites, which include bulky DNA adducts and reactive oxygen species (ROS) generation by cytochrome P-4501A1 (CYP1A1) in mammalian cells [8,9,10]. DNA adducts generated a hot spot in genes and cause the inactivation of tumor suppressor genes or the activation of oncogenes leading to DNA damage, mutation, and cancer [11,12,13]. Furthermore, ROS and metabolites can not only cause oxidative DNA damage, but also produce DNA adducts, which form the mutagenic chain for tumorigenesis [1]. Previous studies reported that these compounds were related to neurotoxicity and resulted in a decrease of neurobehavioral responses [13,14,15]. B[α]P exposure can trigger an increase of malondialdehyde, which is an oxidative stress factor in brain cells [16]. It was revealed that the increase of malondialdehyde induced by B[α]P leads to ROS generation in the brain [17].

The major catechins in green tea, which includes of (−)-epigallocatechin-3-gallate (EGCG), (−)-epigallocatechin gallate (EGCG), (−)-epicatechin gallate (ECG), (−)-epigallocatechin (EGC), and (−)-epicatechin (EC) are well known for protection of chronic diseases, including cancer [18,19]. Several studies previously reported the preventive effect of catechins from green tea on carcinogenesis induced by B[α]P. In detail, green tea catechins were effective to inhibit DNA adducts formation induced by B[α]P, which indicated an interaction of the hydroxyl group in the chemical structure and inhibition activity [20]. Another study also reported that inhibitory effects of catechins on gene expression was related to oxidative stress and the tumor necrosis factor (TNF) in vitro cell culture system [10].

Several studies proved that co-consumption of polyphenol-rich foods reduced bio-accessibility of environmental contaminants by over 90% using the in vitro digestion model [21,22]. Lee et al. (2018) found that the oral intake of quercetin reduced the small intestinal absorption of B[α]P in human intestinal epithelial cells (Caco-2 cells) [23]. However, information about the overall transport mechanism and toxicity to target organs, such as the brain followed by oral administration of B[α]P, is limited. To carry this out, our group previously established a sequential bio-mimic model system that simulated the gastrointestinal tract to the blood-brain barrier (BBB) coupled with Caco-2, human liver hepatocellular carcinoma (HepG2), and human brain microvascular endothelial cells (HBMECs) cell co-cultures and then evaluated the bio-availability of green tea catechins and its health benefits on brain function [24]. Thus, the aims of the current study were to examine the effects of catechins on (1) cell cytotoxicity of hepatocyte and BBB induced by B[α]P using HepG2 and HBMECs cell lines, (2) bio-accessibility of B[α]P using the in vitro digestion model system, and (3) transport of B[α]P using a sequential cell cultures system.

## 2. Results and Discussion

### 2.1. Preventive Effect of Catechins on Cell Cytotoxicity Induced by B[α]P in HepG2 and HBMECs

**Figure 1:**
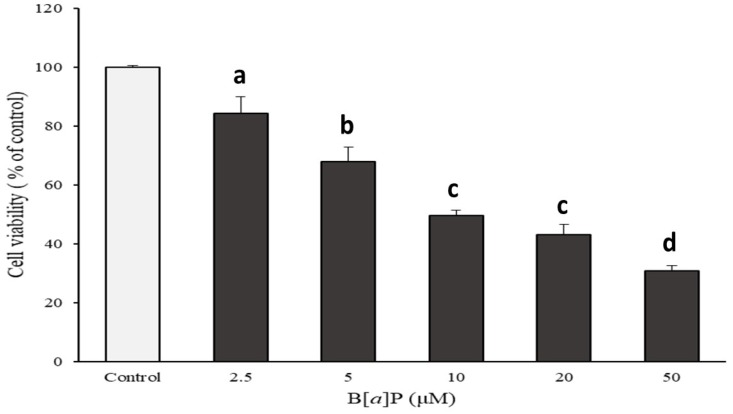
Effects of Benzo[a]pyrene (B[α]P) on cell viability (% of control). human liver hepatocellular carcinoma (HepG2) cells were treated with different concentrations of B[α]P for 24 hr. The values are mean ± SD in triplicate. Each bar with different letters is significantly different. ^a^ *p* < 0.01 as compared with control; ^b^ *p* < 0.001 as compared with control; ^c^ *p* < 0.001 as compared with control; ^d^ *p* < 0.001 as compared with control. Control indicates HepG2 treated by Dulbecco’s Modified Eagle Medium (DMEM) without B[α]P.

**Figure 2:**
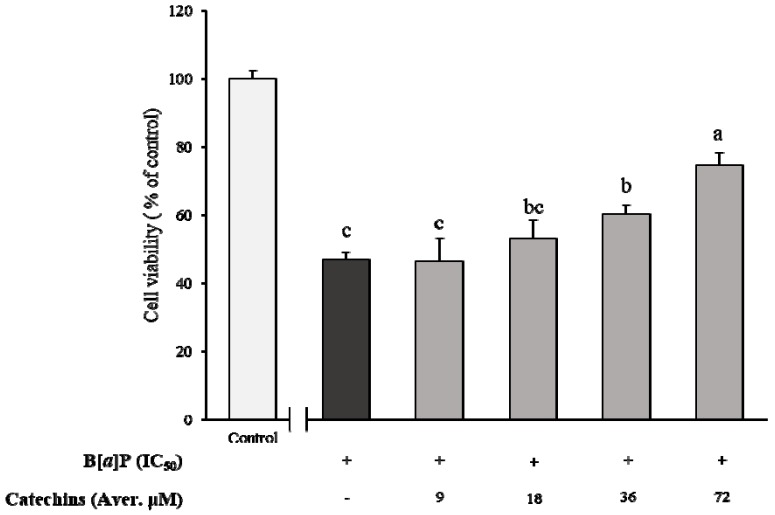
Determination of B[α]P treated HepG2 cell viability (% of control) after co-culture with different concentrations of catechins for 24 h. The values are mean ± SD in triplicate. Each bar with different letters is significantly different among treatments. ^a^ *p* < 0.001 as compared with control; ^b^ *p* < 0.001 as compared with control; ^c^ *p* < 0.001 as compared with control. Control indicates HepG2 cells treated by DMEM without B[α]P.

Cell cytotoxicity of HepG2 cells cultured with various concentrations of B[α]P presented a concentration-dependent pattern, which showed 84, 68, 49, 43, and 30% of cell viability from 2.5, 5, 10, 20, and 50 μM of B[α]P exposure, respectively (Figure 1). Based on the results, the concentration that could inhibit cell growth by 50% (IC_50_) was determined as 10 μM of B[α]P. In order to evaluate the inhibitory effect of catechins on HepG2 cytotoxicity induced by B[α]P, various concentrations of catechins were pre-treated prior to incubation of IC_50_ of B[α]P. The cell viability was 46, 53, 60, and 74% when catechins ranged from 9, 18, 36, and 72 μM, respectively. In the meanwhile, catechins were stable within 24 h of incubation, containing over 90% of remaining catechins. There was a significant difference in the pre-treatment of 37 and 72 μM of catechins from IC_50_ of B[α]P only (*p* < 0.05) (Figure 2). The results from the current study indicated that catechins prevented cell cytotoxicity of HepG2 induced by B[α]P in a concentration-dependent manner.

**Figure 3:**
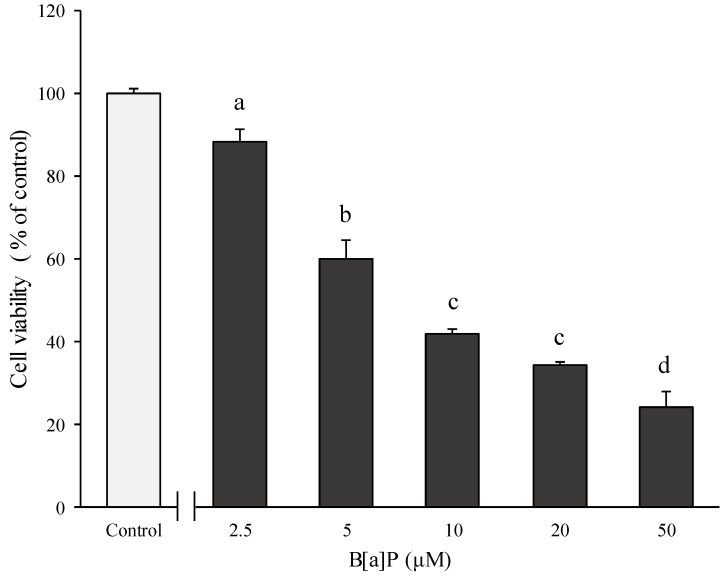
Effects of B[α]P on cell viability (% of control). The human brain microvascular endothelial cells (HBMECs) were treated with different concentrations of B[α]P for 24 h. The values are mean ± SD in triplicate. Each bar with different letters is significantly different. ^a^ *p* < 0.01 as compared with control; ^b^ *p* < 0.001 as compared with control; ^c^ *p* < 0.001 as compared with control; ^d^ *p* < 0.001 as compared with control. Control indicates HBMECs treated by DMEM without B[α]P.

**Figure 4:**
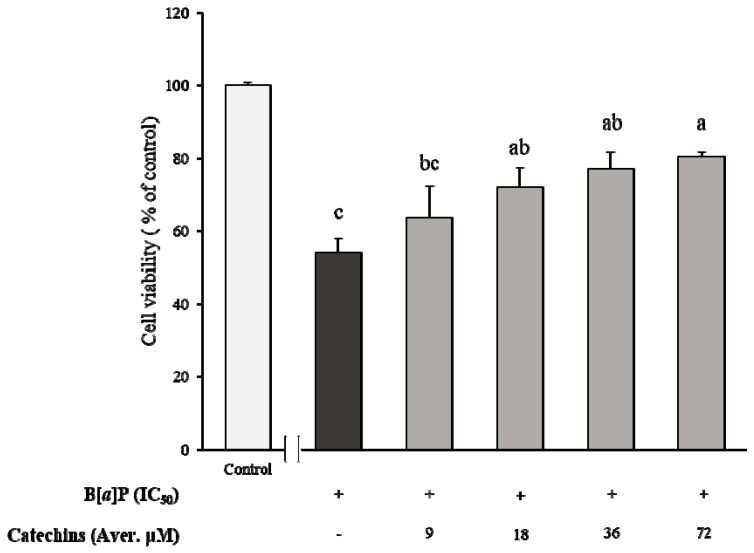
Determination of B[α]P treated HBMECs cell viability (% of control) after co-culture with different concentrations of catechins for 24 h. The values are mean ± SD in triplicate. Each bar with different letters is significantly different among treatments. ^a^ *p* < 0.01 as compared with control; ^b^ *p* < 0.01 as compared with control; ^c^ *p* < 0.001 as compared with control. Control indicates HBMECs treated by DMEM without B[α]P.

Figure 3 shows that the HBMECs cytotoxicity was induced by various concentrations of B[α]P, and it appeared to be in a dose-dependent manner that reached from 24–88% at various concentrations of B[α]P, which ranged from 2.5–50 μM. From this data, a concentration at 7.7 μM of B[α]P was determined as the IC_50_ value for the HBMECs. Afterwards, the preventive effect of catechins on cell cytotoxicity caused by B[α]P was also evaluated after pre-treatments of 9 to 72 μM of catechins followed by incubating IC_50_ of B[α]P for 24 h (Figure 4). In contrast to the HepG2 cell, it was not in a concentration-dependent manner, which had a non-significant effect among the pre-treatments of catechins. When high concentrations of catechins, which included 36 and 72 μM, were pre-treated prior to 24 h, the cell viability was significantly amplified with ranges from 77% to 80% compared to only the B[α]P treatment (*p* < 0.05). However, the cell viability was dramatically attenuated after pre-treatment with the above concentration of 72 μM of catechins (data not shown). When IC_50_ of B[α]P was compared between the HBMECs and the HepG2, the HBMECs (7.7 μM) were slightly sensitive to cytotoxicity induced by B[α]P than HepG2 with 10 μM of IC_50_. It was reported that 5 μM of B[α]P reduced the HepG2 cell viability to about 44% [25]. Another study determined the concentration inhibited the cell growth by 20% (IC_20_) of B[α]P to 8.35 μM by exposing the B[α]P for 48 h to the HepG2 cell [26].

B[α]P produces ROS, and it is metabolized to B[α]P-7, 8-dihydrodiol-9, and 10-epoxide (BPDE) by CYP1A1, which resulted in oxidative DNA damage [8]. However, oxidative DNA damage induced by B[α]P exposure has not been clearly related to cell apoptosis yet. The results from a previous study revealed the induction of DNA damage by B[α]P activated p53, which is a tumor suppressor inducing cell apoptosis [27]. Park et al. (2006) also demonstrated that the increase of p53 could be led to activate p21, which is related to cell cycle interruption [1]. The results suggested that B[α]P could be a factor inducing cell apoptosis. Although the expression of CYP1A1 to require B[α]P metabolism was very low, B[α]P could possibly penetrate the blood-brain barrier, due to its hydrophobicity [13]. It is known to bind with AHR, which is located in the cytoplasm and translocates into the nucleus [28]. Transference of AHR into the nucleus allows activation of the expression of CYP1A1 [13]. In addition, AHR bound with the B[α]P inhibited gene expression of the *N*-methyl-*D*-aspartate-type glutamate receptor (NMDAR) subunits that are responsible for learning and memory formation [13]. There are similar findings that can support results from the current study about the preventing effect of catechins on HepG2 cytotoxicity induced by B[α]P. One of them reported that the inhibitory effect of green tea catechins on CYP1A1 activity was significant in the following descending order: ECG≈EGCG>EC≈EGC [29]. The study suggested that the inhibition of CYP1A1 activity by catechins could constrain the metabolism of B[α]P. A previous study demonstrated that four kinds of epi form of catechins were effective in preventing DNA adducts formation induced by B[α]P especially the galloyl catechins EGCG and ECG, which were highly effective [29]. The inhibition rate of DNA adducts reached 64% for EGCG and 39% for ECG. Palermo et al. (2003) demonstrated that catechins inhibited the AHR activity as its antagonists [30]. In detail, a 50% decline of AHR’s activity with 100 μg/mL of green tea extracts (GTE) was observed, and the AHR antagonist activity was different among catechins: EGCG>ECG>EGC>EC. Kumar et al. (2012) also found that intake of green tea elevated the endogenous antioxidants activity, which was inhibited by B[α]P [31]. Therefore, those studies implied that catechins could be capable of preventing B[α]P toxicity. Thus, the results from the current study could be a useful database about developing natural ingredients regarding preventing brain toxicity induced by B[α]P.

### 2.2. Preventive Effect of Catechins on Cellular Integrity of HBMECs Induced by B[α]P Exposure

**Figure 5:**
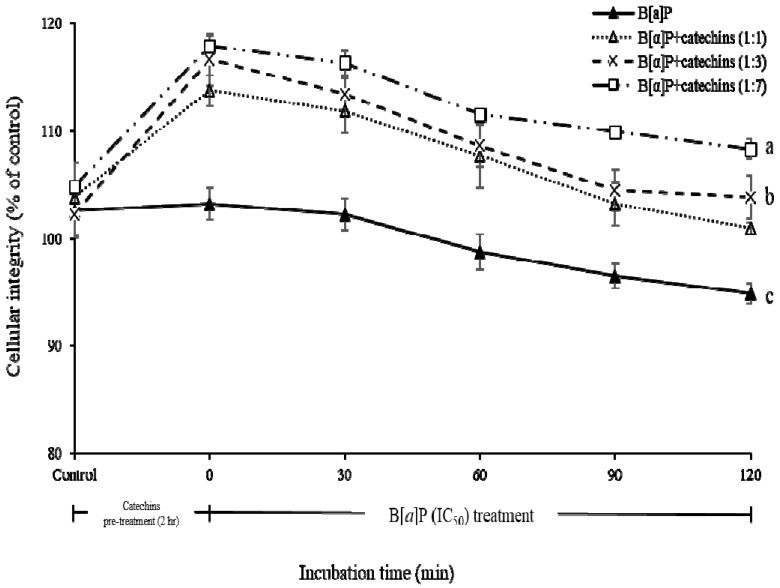
Cellular integrity of HBMECs in B[α]P after pre-treatment with different ratio of catechins. The values are mean ± SD in triplicate. ^a^ *p* < 0.01 as compared with control; ^b^ *p* < 0.05 as compared with control; ^c^ *p* <0.01 as compared with control. Control indicates HBMECs treated by only DMEM without B[α]P and catechins.

The current study evaluated the cellular integrity of HBMECs induced by the IC_50_ value of B[α]P after treatment with different ratios of catechins (B[α]P vs. catechins; 1:0, 1:1, 1:3, 1:7) by measuring the TEER values (Figure 5). When B[α]P was treated without catechins, the cellular integrity slightly and slowly declined according to the incubation time and reduced after 2 h of incubation, and it reached 94% at 120 min. When different ratios of catechins were pre-treated, the cellular integrity increased by approximately 9–14% compared with the initial incubation time. The cellular integrity steadily decreased as incubation time increased after treatment with the IC_50_ value of B[α]P. However, the final cellular integrity of HBMECs treated with catechins was a maximum 1.14 times higher than with the absence of the catechins pre-treatment.

Similar to our findings, Suzuki and Hara 2011 reported that treatment with EGCG increased the TEER value and ameliorated dysfunction of the cellular tight junction barrier [32]. Catechins alleviated the damage of tight junction proteins induced by brain injury and enhanced the BBB integrity [33,34]. Although the B[α]P exposure to brain neuron cells can induce a decrease of learning or memory activity, there were limited studies that evaluated how much of it can be transported to cause toxicity. To clarify the hypothesis that catechins may control absorption of B[α]P in BBB, the current study further investigated the impact of catechins on bioaccessibility, absorption, and transport of B[α]P on the brain by using the in vitro bio-mimic model system coupled with a sequential cell culture that included Caco-2, HepG2, and HBMECs.

### 2.3. Transport of B[α]P from the Gastrointestinal Tract to the Brain by the In Vitro Bio-Mimic Model System Coupled with a Sequential Cell Culture that Included Caco-2, HepG2, and HBMECs

**Figure 6:**
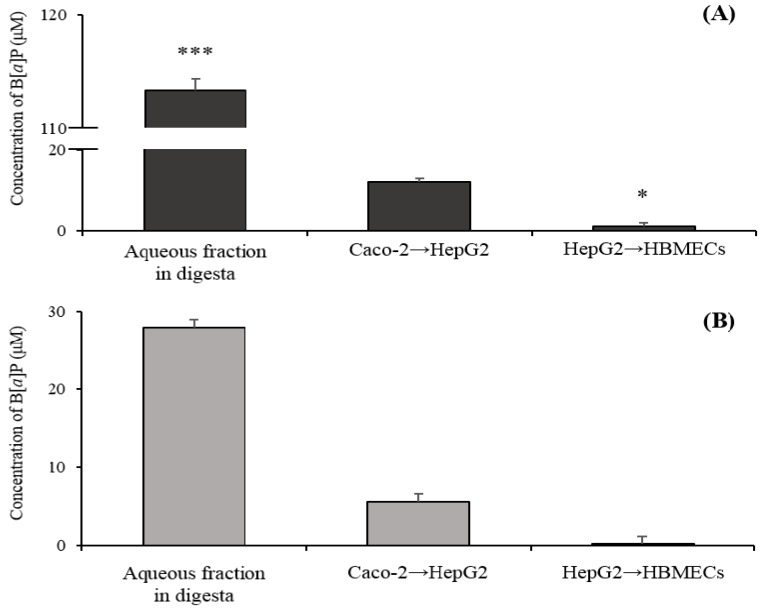
The transported concentration of B[α]P to HBMECs by the in vitro bio-mimic system (Upper GI tract→human intestinal epithelial cells (Caco-2)→HepG2→HBMECs). B[α]P only (**A**) and B[α]P with catechins (**B**). The values are mean ± SD in triplicate. The asterisk indicates a significant difference between groups. (* *p* < 0.05 and *** *p* < 0.001).

According to the results from cell viability in HepG2 and HBMECs, the specific ratio of B[α]P to catechins (1000 μM:7200 μM) was chosen and then co-digested in the gastrointestinal tract to the brain by the in vitro bio-mimic model system coupled with a sequential cell culture that included Caco-2, HepG2, and HBMECs (Figure 6). The concentration of B[α]P in an aqueous fraction obtained after having gone through an artificially simulated gastrointestinal tract was 113.31 ± 11.08 μM of B[α]P, which indicated that 11.33 ± 1.10% of B[α]P remained and was possibly absorbed into the small intestine (Figure 6A). When B[α]P was co-digested with catechins, 27.96 ± 5.93 μM of B[α]P reached into an aqueous fraction of digesta, and its digestive recovery was 2.79 ± 0.59% (Figure 6B). The aqueous fraction of digesta was subsequently dispensed into the co-culture system of Caco-2-HepG2 and then HBMECs to evaluate the total B[α]P to reach the brain after oral intake. After ingestion of B[α]P, the relative amount of B[α]P transported from the Caco-2 (small intestine) to the HepG2 (hepatocyte) was 11.90 ± 4.31 μM, and the final concentration permeated B[α]P from hepatocyte into HBMECs (BBB) was 0.92 ± 0.32 μM (Figure 6A). Meanwhile, co-digestion of B[α]P with catechins absorbed B[α]P from the small intestine to hepatocyte was 5.56 ± 1.05 μM, which was approximately two times lower than only B[α]P digestion. The final concentration of B[α]P transported to BBB was approximately 5.26 times lower than that of only B[α]P digestion, which was 0.17 ± 0.05 μM (Figure 6B).

B[α]P as a lipid-soluble substance should be micellized by bile acid and lipase in digestion fluids in order to be absorbed. For instance, the absorption of B[α]P could be promoted by enzymes, bile acid, and lipase [35]. Lee et al. (2018) reported that the amount of B[α]P in the digestive fluid were 48.7, 48.6, and 46.1% without bile acid, lipase, and bile acid and lipase, respectively, which were similar to our findings. This suggested a significant decrease, and it was presented in comparison with a general digestion condition (66%) [23]. The results from the current study showed that the digestive recovery of B[α]P co-digestion with catechins was five times lower than that of only B[α]P. It is plausible that catechins interrupted the interaction between B[α]P and bile acids or lipase, which limited solubilizing B[α]P. Ogawa et al. (2015) investigated whether the inhibition of micelle formation could be contributed, due to a regiospecific interaction between the gallate group of EGCG and the steroid structure of bile acids [36]. Other studies reported that tea polyphenols that included catechins inhibited 54% of lipase activity, which suggested that tea polyphenols containing the hydroxyl group and the galloyl group might affect the enzyme activity by a configuration change binding of a hydrogen bond and a hydrophobic bond together with an enzyme [37,38]. Results from the current study suggested that oral intake of catechins with B[α]P reduced the absorption of B[α]P in the small intestine, which is shown in Figure 6. B[α]P, which is lipophilic, is able to permeate from intestinal lumen to blood flow by a transcellular transport. Russell (1999) found that the low concentration of B[α]P transport was accelerated at the initial step, but the final transport was reduced [39]. This tendency implies the drug-mediated interaction of intestinal ATP-binding cassette (ABC) transporters. It was reported that B[α]P acted as a substrate of ABC transporters, such as P-gp, MRP2, and BCRP, lead to an increase in efflux transport [40]. Some studies suggested that flavonoids are capable of interfering with the activity of ABC efflux transporters [41]. A previous study suggested that co-consumption of quercetin could contribute to a reduction of B[α]P absorption into the small intestine [23]. Although a number of studies have reported about B[α]P metabolism, studies on bioavailability B[α]P from the gastrointestinal tract to the brain using the in vitro bio-mimic model system coupled with a sequential cell culture are scarce. Results from the current study observed that the amount of B[α]P reaching the brain declined when B[α]P was orally taken with catechins, which implied that co-consumption of catechins are able to prevent a toxic effect of B[α]P in the brain.

## 3. Materials and Methods

### 3.1. Chemicals and Standard Reagents

The analytical standards of (−)-epigallocatechin-3-gallate (EGCG), (−)-epigallocatechin gallate (EGCG), (−)-epicatechin gallate (ECG), (−)-epigallocatechin (EGC), and (−)-epicatechin (EC) were purchased from Wako (Osaka, Japan). Benzo[α]pyrene (B[α]P), α-amylase from human saliva, pepsin, from porcine gastric mucosa, bile extract from porcine, lipase from porcine pancreas, and pancreatin from porcine pancreas were obtained from Sigma Aldrich (St. Louis, MO, USA). Water, methanol, acetonitrile, and acetic acid with a grade for ultra-performance liquid chromatography (UPLC) were purchased from J.T. Baker (Phillipsburg, NJ, USA.) and Sigma Aldrich (St. Louis, MO, USA), respectively. Sodium acetate, dichloromethane (DCM), and ethyl acetate were obtained from Samchun Chemicals (Seoul, Korea).

### 3.2. Cell Cultures

Human intestinal epithelial cells (Caco-2), human liver hepatocellular carcinoma (HepG2), and human brain microvascular endothelial cells (HBMECs) were obtained from the Korean Cell Line Bank (KCLB, Seoul, Republic of Korea), American Type Culture Collection (ATCC, Manassas, VA, USA), and Johns Hopkins University (Baltimore, MD, USA), respectively. Both cells were maintained in DMEM with 10% FBS and 1% Pen/Strep in a humidified atmosphere with 5% CO_2_ and 95% air at 37 °C. The cell media was changed every 2–3 days and sub-cultured at 70% to 80% of cell confluence.

### 3.3. Measurement of Cell Viability

Cell viability was performed on the mono-culture incubated with B[α]P using a 3-(4-5-dimethylthiazol-2-yl)-2.5-dyphenyltetrazolium bromide (MTT) assay (Sigma Aldrich, St. Louis, MO, USA). HepG2 cell (2.4 × 10^4^/well) or HBMECs (5.0 × 10^3^/well) was seeded in 96 well plates (SPL life Science, Pocheon, Republic of Korea). After incubation for 24 h, the cell was treated with various concentrations (2.5, 5, 10, 20, and 50 μM) to determine a concentration that inhibited cell growth by 50% (IC_50_). To evaluate the non-cytotoxic concentration ranges of catechins, various concentrations of the epi-catechins mixtures (9, 18, 36, and 72 μM), which were 1 to 1, 1 to 2, 1 to 5 ratio of IC_50_ B[α]P to catechins were added into cells and then incubated for 24 h. Then, the determined IC_50_ value of B[α]P was added into each cell. A 100 µL of MTT solution was added to each cell followed by dispersing medium. With the same conditions described above, a reaction was carried out after a 4 h incubation under darkness. After removal of the supernatants, dimethyl sulfoxide (DMSO) was added to each well to dissolve the resultant formazan crystals. Optical density (O.D.) was measured at 570 nm using a microplate reader (Thermo Scientific, San Jose, CA, USA). The experiment was conducted in triplicate, and the percentage of cell viability was calculated according to a previous study [25].

### 3.4. Transport of B[α]P from the Gastrointestinal Tract to the Brain by the In Vitro Bio-Mimic Model System Coupled with Caco-2 Cell, HepG2, and HBMECs

The bio-accessibility of B[α]P or B[α]P with epi-catechins was measured by the in vitro digestion model system from a previous study [24] with slight modifications. Digestive enzymes were stored in ice at 4 °C. Each sample was dissolved with DMSO and diluted with a 20 mM phosphate buffer (PB). For the salivary phase, α-amylase (1 unit/mL) was added in the sample and incubated for 3 min in a shaking water bath at 200 rpm at 37 °C. In the gastric phase, pepsin (40 mg/mL 0.1 M HCl) was added and the pH was adjusted to 2.0 ± 0.1 with 1 M HCl. After that, it was incubated for 1 h with the conditions mentioned above. In the small intestinal phase, the pH increased to 5.3 ± 0.1 with 1 M NaHCO_3_ and pancreatin (2 mg/mL PB), lipase (1 mg/mL PB), and bile acid (12 mg/mL PB) were added. The final pH was adjusted to 7.0 ± 0.1 with 1 M NaOH and incubated for 2 h with 200 rpm at 37 °C in a water bath. The total volume of all samples was adjusted equally with a 20 mM PB. The supernatants were collected for the cell treatments and further analysis by ultra-performance liquid chromatography (UPLC).

For the in vitro bio-mimic system, Caco-2 cells (1.0 × 10^5^/well) and HBMECs (1.0 × 10^5^/well) were seeded on polytetrafluoroethylene (PETE)/collagen-coated transwell inserts (12 mm diameter, 0.4 μm pore size, Corning Costar^®^, New York, NY, USA) and put in 12 well plates. A HepG2 cell (1.5–3.0 × 10^5^/well) was seeded on the basolateral side of the 12 well plates. A co-culture system of Caco-2 with HepG2 was performed when the trans-epithelial electronic resistance (TEER) value of the Caco-2 cells was more than 200 Ω cm^−2^ and when the cell confluence of HepG2 reached 70% to 80%. The digestive aqueous fractions of B[α]P or B[α]P with epi-catechins were treated to the apical side of the the Caco-2-HepG2 co-culture system and incubated for 1 h. The basolateral media was collected and then dispensed into the apical side of the HBMECs, which the TEER values were over 100 Ω cm^−2^. After a 1 h incubation, the basolateral media were used for further analysis by the UPLC. All experiments were conducted in triplicate.

### 3.5. Analysis of B[α]P by UPLC

For extraction of B[α]P, the samples were dissolved in a sodium acetate buffer (0.2 M, pH 4). The samples were mixed with acetone and then extracted with ethyl acetate three times. The ethyl acetate extract was dehydrated with anhydrous sodium sulfate, as well as evaporated by a nitrogen evaporator (Organomation, Berlin, MA, USA) at a temperature below 40 °C. After that, the dry residue was dissolved in hexane before solid phase extraction through a Sep-Pak^®^ Vac 6 cc (1 g) C18 cartridges (Waters, Milford, MA, USA) with DCM and hexane. The organic solvents were completely evaporated under nitrogen gas at a temperature below 40 °C. For the analysis, dry residue samples were dissolved in ACN and filtered with a PVDF 0.2 μm syringe filter. The chromatography analysis was conducted using an Ultimate 3000 UPLC (Thermo Fisher Scientific, Waltham, MA, USA) with a reversed phase column (Zorbax Eclipse XDB C18 column 4.6 × 250 mm, 5 μm, Agilent Technologies, Santa Clara, CA, USA). The mobile phase was ACN (mobile phase A) and water (mobile phase B). The gradient elution was 80% A + 20% B at 0–20 min, 100% A at 20–25 min, and 80% A + 20% B at 25–30 min with a flow rate of 1.0 mL/minute. The injection volume and UV wavelength was 20 μL and 280 nm, respectively.

### 3.6. Statistical Analysis

The values are reported as mean ± standard deviation (SD). All experiments were conducted in triplicate. One-way analysis of variance (ANOVA) and Tukey’s post hoc test was carried out to verify significant differences among the groups at a significance level of *p* < 0.05 using Graphpad Prism software (Ver. 3.0, San Diego, CA, USA).

## 4. Conclusions

The current study evaluated the effect of green tea catechins on the cytotoxicity of hepatocyte and the blood-brain barrier using HepG2 and HBMECs, respectively. Control of B[α]P bioavailability by catechins after oral intake from the gastrointestinal tract to the brain was conducted using the in vitro bio-mimic model system, which included the sequential cell cultures system, was evaluated. Catechins effectively contributed to enhancing cellular viability and BBB integrity, which was reduced by B[α]P exposure. The absorption of B[α]P by Caco-2 co-cultured with HepG2 cells was decreased with the co-consumption of catechins, which resulted in a decrease of transport of B[α]P into HBMECs. The results suggested that co-consumption of catechins could prevent a toxic effect induced by B[α]P on the brain by limiting the bioavailability of B[α]P.

## References

[1] Park S.-Y., Lee S.-M., Ye S.-K., Yoon S.-H., Chung M.-H., Choi J. (2006). Benzo [a] pyrene-induced DNA damage and p53 modulation in human hepatoma HepG2 cells for the identification of potential biomarkers for PAH monitoring and risk assessment. Toxicol. Lett..

[2] Smith C., Perfetti T., Rumple M., Rodgman A., Doolittle D. (2000). “IARC group 2A Carcinogens” reported in cigarette mainstream smoke. Food Chem. Toxicol..

[3] Kazerouni N., Sinha R., Hsu C.-H., Greenberg A., Rothman N. (2001). Analysis of 200 food items for benzo [a] pyrene and estimation of its intake in an epidemiologic study. Food Chem. Toxicol..

[4] Perera F., Tang D., Whyatt R., Lederman S.A., Jedrychowski W. (2005). DNA damage from polycyclic aromatic hydrocarbons measured by benzo [a] pyrene-DNA adducts in mothers and newborns from Northern Manhattan, the World Trade Center Area, Poland, and China. Cancer Epidem. Biomar..

[5] Stumpe-Viksna I., Morozovs A., Bartkevics V., Kukare A. (2008). Levels of benzo (a) pyrene (BaP) in fish, smoked according to different procedures. Proc. Latvia Univ. Agric..

[6] Lee B.M., Shim G.A. (2007). Dietary exposure estimation of benzo[a]pyrene and cancer risk assessment. J. Toxicol. Environ. Health A.

[7] (2006). Joint FAO/WHO Expert Committee. Evaluation of certain food contaminants: Sixty-fourth report of the Joint FAO/WHO Expert Committee on Food Additives. https://apps.who.int/iris/bitstream/handle/10665/43258/WHO_TRS_930_eng.pdf.

[8] Burczynski M.E., Penning T.M. (2000). Genotoxic polycyclic aromatic hydrocarbon ortho-quinones generated by aldo-keto reductases induce CYP1A1 via nuclear translocation of the aryl hydrocarbon receptor. Cancer Res..

[9] Miller K.P., Ramos K.S. (2001). Impact of cellular metabolism on the biological effects of benzo[a]pyrene and related hydrocarbons. Drug Metab. Rev..

[10] Omidian K., Rafiei H., Bandy B. (2017). Polyphenol inhibition of benzo[a]pyrene-induced oxidative stress and neoplastic transformation in an in vitro model of carcinogenesis. Food Chem. Toxicol..

[11] Baird W.M., Hooven L.A., Mahadevan B. (2005). Carcinogenic polycyclic aromatic hydrocarbon-DNA adducts and mechanism of action. Environ. Mol. Mutagen..

[12] Nesnow S., Nelson G., Padgett W.T., George M.H., Moore T., King L.C., Adams L.D., Ross J.A. (2010). Lack of contribution of covalent benzo[a]pyrene-7,8-quinone-DNA adducts in benzo[a]pyrene-induced mouse lung tumorigenesis. Chem Biol Interact.

[13] Chepelev N.L., Moffat I.D., Bowers W.J., Yauk C.L. (2015). Neurotoxicity may be an overlooked consequence of benzo[a]pyrene exposure that is relevant to human health risk assessment. Mutat. Res. Rev. Mutat. Res..

[14] Wormley D.D., Ramesh A., Hood D.B. (2004). Environmental contaminant-mixture effects on CNS development, plasticity, and behavior. Toxicol. Appl. Pharmacol..

[15] Grova N., Valley A., Turner J.D., Morel A., Muller C.P., Schroeder H. (2007). Modulation of behavior and NMDA-R1 gene mRNA expression in adult female mice after sub-acute administration of benzo(a)pyrene. Neurotoxicology.

[16] Saunders C.R., Das S.K., Ramesh A., Shockley D.C., Mukherjee S. (2006). Benzo(a)pyrene-induced acute neurotoxicity in the F-344 rat: Role of oxidative stress. J. Appl. Toxicol..

[17] Dutta K., Ghosh D., Nazmi A., Kumawat K.L., Basu A. (2010). A common carcinogen benzo[a]pyrene causes neuronal death in mouse via microglial activation. PLoS ONE.

[18] Vauzour D. (2012). Dietary polyphenols as modulators of brain functions: Biological actions and molecular mechanisms underpinning their beneficial effects. Oxid Med Cell Longev.

[19] Ananingsih V.K., Sharma A., Zhou W. (2013). Green tea catechins during food processing and storage: A review on stability and detection. Food Res. Int..

[20] Cao P., Cai J., Gupta R.C. (2010). Effect of Green Tea Catechins and Hydrolyzable Tannins on Benzo [a] pyrene-Induced DNA Adducts and Structure− Activity Relationship. Chem. Res. Toxicol..

[21] Shim S.-M., Ferruzzi M.G., Kim Y.-C., Janle E.M., Santerre C.R. (2009). Impact of phytochemical-rich foods on bioaccessibility of mercury from fish. Food Chem..

[22] Girard C., Charette T., Leclerc M., Shapiro B.J., Amyot M. (2018). Cooking and co-ingested polyphenols reduce in vitro methylmercury bioaccessibility from fish and may alter exposure in humans. Sci. Total Environ..

[23] Truong N.-H., Lee S., Shim S.-M. (2016). Screening bioactive components affecting the capacity of bile acid binding and pancreatic lipase inhibitory activity. Appl. Biol. Chem..

[24] Jeong K.-H., Cho S.-Y., Hong Y.-D., Chung J.-O., Kim K.-S., Shim S.-M. (2018). Transport of gallocatechin gallate and catechin gallate in high-temperature-processed green tea extract from gastrointestinal tract to brain by an in vitro bio-mimic model system coupled with sequential cell cultures. J. Funct. Foods.

[25] Lee S.B., Kim J.H., Cho M.H., Choe E.S., Kim K.S., Shim S.M. (2017). Impact of commercial cigarette smoke condensate on brain tissue co-cultured with astrocytes and blood-brain barrier endothelial cells. J. Toxicol. Environ. Health A.

[26] Song M.-K., Yoon J.-S., Song M., Choi H.-S., Shin C.-Y., Kim Y.-J., Ryu W.-I., Lee H.-S., Ryu J.-C. (2012). Gene expression analysis identifies DNA damage-related markers of benzo[a]pyrene exposure in HepG2 human hepatocytes. Toxicol. Env. Health Sci..

[27] Ueno M., Masutani H., Arai R.J., Yamauchi A., Hirota K., Sakai T., Inamoto T., Yamaoka Y., Yodoi J., Nikaido T. (1999). Thioredoxin-dependent redox regulation of p53-mediated p21 activation. J. Biol. Chem..

[28] Ovesen J.L., Schnekenburger M., Puga A. (2011). Aryl hydrocarbon receptor ligands of widely different toxic equivalency factors induce similar histone marks in target gene chromatin. Toxicol. Sci..

[29] Muto S., Fujita K.-i., Yamazaki Y., Kamataki T. (2001). Inhibition by green tea catechins of metabolic activation of procarcinogens by human cytochrome P450. Mutat. Res.-Fund. Mol. Mech. Mutagenesis.

[30] Palermo C., Hernando J.M., Dertinger S., Kende A., Gasiewicz T. (2003). Identification of potential aryl hydrocarbon receptor antagonists in green tea. Chem. Res. Toxicol..

[31] Kumar M., Sharma V., Sehgal A., Jain M. (2012). Protective effects of green and white tea against benzo(a)pyrene induced oxidative stress and DNA damage in murine model. Nutr. Cancer.

[32] Suzuki T., Hara H. (2011). Role of flavonoids in intestinal tight junction regulation. J. Nutr. Biochem..

[33] Bhardwaj P., Khanna D. (2013). Green tea catechins: Defensive role in cardiovascular disorders. Chin. J. Nat. Med..

[34] Jiang Z., Zhang J., Cai Y., Huang J., You L. (2017). Catechin attenuates traumatic brain injury-induced blood-brain barrier damage and improves longer-term neurological outcomes in rats. Exp. Physiol..

[35] Ogawa K., Hirose S., Nagaoka S., Yanase E. (2015). Interaction between tea polyphenols and bile acid inhibits micellar cholesterol solubility. J. Agric. Food Chem..

[36] He Q., Shi B., Yao K. (2006). Interactions of gallotannins with proteins, amino acids, phospholipids and sugars. Food Chem..

[37] He Q., Lv Y., Yao K. (2007). Effects of tea polyphenols on the activities of α-amylase, pepsin, trypsin and lipase. Food Chem..

[38] Russell M. (1999). In Vitro Model for Intestinal Uptake of benzo[a]pyrene. https://escholarship.org/uc/item/5k06t6rr.

[39] Brand W., Schutte M.E., Williamson G., van Zanden J.J., Cnubben N.H., Groten J.P., van Bladeren P.J., Rietjens I.M. (2006). Flavonoid-mediated inhibition of intestinal ABC transporters may affect the oral bioavailability of drugs, food-borne toxic compounds and bioactive ingredients. Biomed. Pharmacother..

[40] Romiti N., Tramonti G., Donati A., Chieli E. (2004). Effects of grapefruit juice on the multidrug transporter P-glycoprotein in the human proximal tubular cell line HK-2. Life Sci..

[41] Zhang S., Morris M.E. (2003). Effect of the flavonoids biochanin A and silymarin on the P-glycoprotein-mediated transport of digoxin and vinblastine in human intestinal Caco-2 cells. Pharm. Res..

